# Concentration of Strontium-90 at Selected Hot Spots in Japan

**DOI:** 10.1371/journal.pone.0057760

**Published:** 2013-03-07

**Authors:** Georg Steinhauser, Viktoria Schauer, Katsumi Shozugawa

**Affiliations:** 1 Department of Environmental and Radiological Health Sciences, Colorado State University, Fort Collins, Colorado, United States of America; 2 Vienna University of Technology, Atominstitut, Vienna, Austria; 3 Graduate School of Arts and Sciences, The University of Tokyo, Meguro-ku, Tokyo, Japan; University of Zurich, Switzerland

## Abstract

This study is dedicated to the environmental monitoring of radionuclides released in the course of the Fukushima nuclear accident. The activity concentrations of β^−^ -emitting ^90^Sr and β^−^/γ-emitting ^134^Cs and ^137^Cs from several hot spots in Japan were determined in soil and vegetation samples. The ^90^Sr contamination levels of the samples were relatively low and did not exceed the Bq⋅g^−1^ range. They were up four orders of magnitude lower than the respective ^137^Cs levels. This study, therefore, experimentally confirms previous predictions indicating a low release of ^90^Sr from the Fukushima reactors, due to its low volatility. The radiocesium contamination could be clearly attributed to the Fukushima nuclear accident via its activity ratio fingerprint (^134^Cs/^137^Cs). Although the correlation between ^90^Sr and ^137^Cs is relatively weak, the data set suggests an intrinsic coexistence of both radionuclides in the contaminations caused by the Fukushima nuclear accident. This observation is of great importance not only for remediation campaigns but also for the current food monitoring campaigns, which currently rely on the assumption that the activity concentrations of β^−^-emitting ^90^Sr (which is relatively laborious to determine) is not higher than 10% of the level of γ-emitting ^137^Cs (which can be measured quickly). This assumption could be confirmed for the samples investigated herein.

## Introduction

In the course of the Fukushima nuclear accident, large amounts of volatile radionuclides were released into the environment. In particular, the radioisotopes of xenon, krypton, iodine (especially ^131^I), cesium (especially ^134^Cs and ^137^Cs), and tellurium are regarded as the most relevant ones, which caused partly significant contamination of the Japanese land surface [1]–[3] and the Pacific Ocean [4]–[9]. These radionuclides were monitored globally in various environmental media, see e.g. [10]–[16]. Few studies also indicated the release of low amounts of less volatile radionuclides, such as ^59^Fe, ^95^Nb, ^140^Ba, ^140^La, ^239^Np and many others [17].

What all the above mentioned radionuclides have in common is that they are γ-emitters, which allows their straightforward detection and quantification using γ-spectrometry. The analysis of pure β^−^ -emitters, in contrast, requires greater efforts. This also refers to radiostrontium, in particular ^90^Sr (T_1/2_ = 28.90 yr). Due to its chemical similarity to calcium, ^90^Sr is accumulated in the bone and may cause leukemia or skeletal cancer. Its presence in the environment, therefore, causes much concern as it is often dictating risk of contaminated sites over longer periods of time and calls for the monitoring of this inconvenient radionuclide [18]. This is of great importance especially for ensuring food safety.

Apart from sea water [19], data base publications [20], [21] and governmental and/or industrial analyses [22]–[24], hardly any data for ^90^Sr released during the Fukushima nuclear accident were published in peer-reviewed literature. Measurements of airborne radiostrontium have been conducted by European networks but did not reveal detectable activities that could be attributed unambiguously to the releases of the Fukushima nuclear accident (e.g. via the presence of short-lived ^89^Sr; T_1/2_ = 50.5 d) [12]. In some cases detectable levels of ^90^Sr were reported for seawater around the Korean Peninsula [25]. The present study is one of the first ones published in peer-reviewed English literature dedicated to the ^90^Sr contamination levels on the Japanese land surface.

## Methods

### Samples

**Figure 1:**
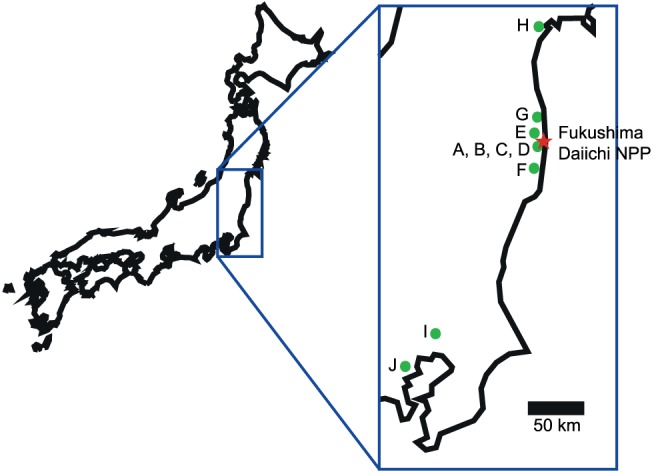
Geographical setting of the sampling sites.

Samples were taken between December 2011 and July 2012 from several spots on the eastern Japanese coast (Fig. 1). All samples were taken from public areas, hence no permits from private land owners were necessary. Apart from the sampling in the restricted areas (for which permission was obtained from the mayor of Ohkuma town), no specific permits were required for the described field studies. Some of the spots have already been investigated previously with respect to short-lived γ-emitting radionuclides [17]. On most sites two types of samples were taken: soil and vegetation (leaves, grass, conifer needles etc.). The studies did not involve protected or endangered species. The sampling sites included highly contaminated spots such the gate of the Fukushima Daiichi nuclear power plant (NPP), locations in the close vicinity of the NPP, but also more remote sites such as Kashiwa (some 200 km south of the NPP). For exact locations and sampling dates, please see Table S1.

### Strontium separation and measurement

Liquid scintillation counting (LSC) was applied for the radiometric analysis of ^90^Sr. It requires a chemically pure strontium sample in order to avoid contributions to the counting signal by other radionuclides in the sample.

It was assumed that Fukushima fallout ^90^Sr was adsorbed on the surface of the soil particles and not immobilized, e.g. by incorporation into the crystals of the mineral components of the soil. Complete dissolution of the soil samples was hence not deemed necessary. In order to not immobilize the surface- adsorbed radiostrontium, the samples were not dried by heating, but carefully air dried in a desiccator. Before radiostrontium extraction, the activity concentrations of ^134^Cs and ^137^Cs were determined by γ-spectrometry to allow for the investigation of the correlation between radiostrontium and radiocesium contamination levels. Hereto, the samples were weighed into a fixed geometry (sample vials with a defined filling height) and measured in a calibrated position with an HPGe γ-spectrometer prior to the extraction procedure (see [26], [27] for detector details).

Strontium was extracted from the soil and vegetation materials using the following procedure. The samples were treated with a mixture of 2.5 mL concentrated HNO_3_ (65%; Roth®, p.a.), 2.5 mL of an aqueous Sr carrier solution (c_Sr_ = 1.2 mg⋅mL^−1^ stable Sr in distilled H_2_O; Sr(NO_3_)_2_ by Riedel de Haen®, extra pure) and 1 mL H_2_O_2_ (30%; Roth®, p.a.). If necessary, additional HNO_3_ and H_2_O_2_ were added during the procedure (which was necessary especially with the plant samples for complete dissolution). The samples were stirred and heated to boil under the reflux of a cooler for 30 min. The mixture was filtered using Schleicher & Schuell® 595 filters and washed with several mL of 8 M HNO_3_. The yellow filtrate was collected and steamed off to dryness. The yellow color, in part, proved to be Fe(III) compounds. The residue was taken up in 4 mL 8 M HNO_3_ and stirred until complete dissolution.

For the separation of radiostrontium from other radionuclides, strontium selective SR-resin-B (100–150 µm) by Eichrom®/Triskem International® was used. The columns for this ion chromatography were produced by Carlo Erba® and fulfill Euratom standards. An amount of 380–420 mg fresh Sr resin was equilibrated in 8 M HNO_3_ at least 90 min before loading with the sample extract onto the column. After loading, the flask was rinsed four times with 0.5 mL 8 M HNO_3_. This rinsing solution was also loaded onto the column. Then the column was rinsed five times with 1 mL of a solution of 3 M HNO_3_ and 0.05 M oxalic acid (Merck®, p.a.). In this washing procedure the yellow color could be removed from the column to the greatest extent. Subsequent elution of Sr was performed with ten times 1 mL of 0.05 M HNO_3_. Any residual yellow color on the column was also retained by the column during the elution, providing a clear and colorless product, which is important for LSC (avoiding color quenching). The product was collected in a flask, evaporated to dryness and taken up in four times 1 mL of 0.01 M HNO_3_ that were pipetted into the LSC counting vials sequentially for the sake of rinsing the flask. Sixteen mL of Ultima Gold™ LSC cocktail (Perkin Elmer®) were added to the 4 mL of aqueous Sr extract, which proved to be the optimum ratio between aqueous phase and LSC cocktail in our case.

The measurements were conducted with a Packard® Tri-Carb 2700TR Liquid Scintillation Analyzer. Each measurement lasted for 1000 min. The samples were measured twice, once immediately after the chemical separation, once after at least two weeks, i.e. after ingrowth of the daughter nuclide ^90^Y (T_1/2_ = 64.1 h) into radioactive equilibrium with its mother ^90^Sr.

The efficiency of the Sr extraction (i.e., the total Sr recovery after dissolution and extraction) was determined using a 3 mg Sr aliquot of a solution of Sr(NO_3_)_2_, which was activated in the Atominstitut's TRIGA Mark II reactor. The resulting ^85^Sr tracer was pipetted onto an aliquot of approximately 200 mg of each sample and carefully dried at 55°C. Then the procedure of Sr extraction was performed as described above. The γ-emitting radioisotope ^85^Sr (T_1/2 = _64.9 d; γ-line at 514 keV) allows straightforward quantification before and after the procedure using γ-spectrometry. The utilization of ^85^Sr is regarded as the most suitable technique for the quantification of the strontium extraction yield in this type of radioecological study [28]. The recoveries were in the range of 92.4–98.2%. They were used to extrapolate the results of the analyses to the total activity concentration of ^90^Sr in the respective samples.

For ^90^Sr quantification in the LSC, IAEA-373 (Radionuclides in Grass) was used as a reference material. The standard's grass matrix was treated in the identical way like the vegetation samples in order to obtain a pure Sr fraction. All nuclear data in this paper are taken from the National Nuclear Data Center [29].

## Results and Discussion

**Figure 2:**
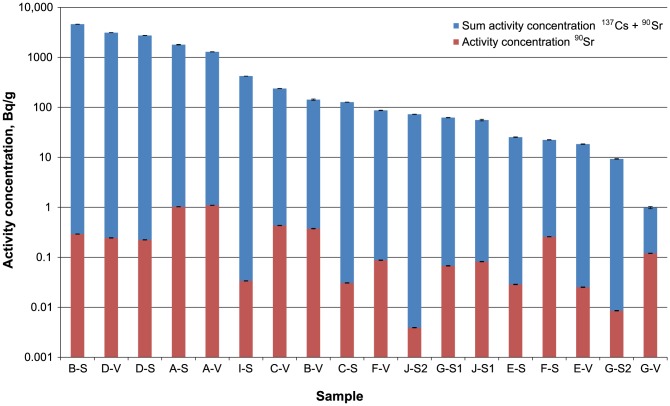
Activity concentrations of ^90^Sr and sum activity concentrations of ^90^Sr + ^137^Cs at the investigated spots. Sample codes include the code of the sampling locations (see Fig. 1) and the type of sample material, soil (S) or vegetation (V). At the spots G and J, two soil samples were taken (indicated by 1 and 2). Error bars are due to counting statistics. Activities were decay corrected to the time of the accident. Please note the logarithmic scale.

The results of the measurements are illustrated in Fig. 2 and tabulated in Table S1. The ^90^Sr levels were generally relatively low (up to approximately 1 Bq⋅g^−1^), and 1 to 4 orders of magnitude lower than the ^137^Cs levels in the respective sample. This experimentally confirms the modeling by Schwantes and colleagues [18], who predicted only very low releases of radiostrontium via the gas phase. In general, a contamination of the land surface occurs via dry deposition of radionuclides, but it is much enhanced through rainfall causing wet deposition. Southern wind directions and rainfall explain the relatively high activity levels in the remote hot spot in Kashiwa (spot code I) and Yokohama (spot code J), which are located close to Tokyo. Accordingly, also local environmental conditions seem to be responsible for the surprisingly low contamination levels at spots E and G that are located not only quite close to the damaged reactors of Fukushima NPP (distance 8.7 and 16.4 km, respectively), but also in or next to the highly contaminated strip in (north)northwestern direction from the NPP that, even outside the restricted area (20 km radius), had been declared as “deliberate evacuation area” [3]. In this special case, probably the local lack of rainfall as well as a specific soil composition led to low contamination levels at spots E and G and/or prevented the retention of both radiostrontium and radiocesium in the top soil at these locations. Our results once again evidence that distance from the source alone is no sufficient factor for the prediction of a contamination level at a certain spot after a nuclear accident.

One has to take into account that pre-Fukushima events (mainly atmospheric nuclear explosions of the 20^th^ century as well as previous nuclear accidents) also contribute to the inventory of both radionuclides ^137^Cs and ^90^Sr. In case of radiocesium, the presence of the shorter-lived reactor nuclide ^134^Cs allows the assignment to the Fukushima accident. Any ^134^Cs from previous nuclear accidents typically has decayed below the limit of detection in environmental samples due to its relatively short half-life (T_1/2_ = 2.07 yr). Previous studies on the Fukushima nuclear accident indicated an activity ratio of ^134^Cs/^137^Cs of approximately 1 or just below 1 at the time of the accident (11 March 2011) [12], [30], which also was found in the present study (see Table S1).

**Figure 3:**
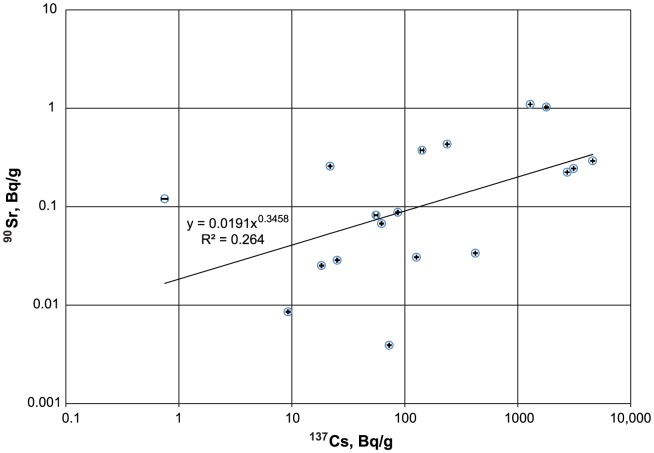
Correlation of the activity concentrations of ^90^Sr vs. ^137^Cs. Error bars are due to counting statistics. Activities were decay corrected to the time of the accident. Please note the logarithmic scales.

In addition to absolute contamination data for ^90^Sr, a possible correlation between the ^90^Sr and radiocesium levels is worth more in-depth discussion. In the ideal case, this should make a rough estimation of the activity concentration of ^90^Sr possible via the straightforward γ-spectrometric determination of ^137^Cs, just as it has been proposed by Japanese authorities with respect to food safety [31]. The authorities assumed intrinsic co-existence of both radionuclides in the environment (including foodstuffs) as well as a maximum ^90^Sr activity of 10% of the respective ^137^Cs activity. The correlations between the activity concentration data of ^90^Sr and ^137^Cs in this study are shown in Fig. 3. Although this correlation is quite weak, a certain trend between the both radionuclides can be observed in this figure. Previous studies showed a reliable correlation of the concentrations of isotopes of the same element, e.g. ^134^Cs/^137^Cs [30], ^136^Cs/^137^Cs [18], or (in a different topic) ^239,240^Pu/^238^Pu [32], which even allowed source identification.

Radionuclide correlations between two different elements always involve problematic factors such as different volatilities in the course of the release as well as chemical fractionation in the environment after the release. The above correlation between ^90^Sr and ^137^Cs hence does not provide more information than a rough estimation of the maximum ^90^Sr activity concentration via the respective ^137^Cs activity concentration, as suggested by the Japanese authorities [31]. Also, one has to take into account, that even after deposition this correlation cannot be regarded as constant over time due to different diffusion, adsorption, and washout behavior of the two elements Cs and Sr. This fact also becomes obvious from Fig. 2, indicating that the relative contamination levels of soil and vegetation at one spot are not always fully comparable. Both soil and vegetation samples from spot B, for example, exhibited a comparable ^90^Sr activity concentration, whereas the ^137^Cs concentration in this location is more than an order of magnitude higher in soil than with the vegetation sample. For a long-term assessment of the radiological risk, the effective ecological half-lives of radiostrontium and radiocesium will have to be determined in the various types of soil in the next years.

Although the correlation proved to be weak (as reflected by the R-squared value of 0.264), the power trend line was found to fit the data set presented in Fig. 3 best. Please note that the limited data set introduces a considerable uncertainty. The relative ^90^Sr activity concentration in highly contaminated areas in close vicinity to the reactors showed to be higher than in more remote areas. It is likely that both dry and wet deposition of less volatile elements such as Sr is enhanced compared with the more volatile Cs. Therefore, it is reasonable to predict that the more remote hot spots northwest of the NPP site will exhibit lower relative ^90^Sr contributions to the total activities than the hot spots next to the reactors. To a minor extent, distinct features of the four different sources (i.e. the four reactors) may be partly responsible for the poor correlation, as characteristics such as the type of fuel, the burn-up rate, the type of damage, as well as the temperature and coolant profiles inside the damaged reactors may have influenced the releases from the four distinct sources [18]. The present data set, however, does not allow identification of these sources in distinct areas as other mechanisms such as chemical fractionation in the environment has concealed such minute differences.

As indicated above, some uncertainty could also have been introduced by the contributions of previous fall-out from nuclear explosions that affects low-contaminated samples to a greater extent than highly contaminated spots. For instance, this could be the case with sample G-V, which has a relatively high ^90^Sr content, whereas the radiocesium content is relatively low. Although the ^134^Cs/^137^Cs activity ratio (0.97) tells us that the radiocesium in this sample is virtually completely due to Fukushima fallout, we can hypothesize that the (low) radiostrontium contamination, in part, could be old fall-out ^90^Sr that has been retained in the soil and taken up by the plant (whereas any old radiocesium may not have been retained by the soil as efficiently). If this sample G-V is omitted in Fig. 3, the R-squared of the resulting power trend line (y = 0.0066 x^0.5204^) “improves” to a value of 0.41, which is still not comparable to the neat correlations between radionuclides of the same element, though.

The presence of bone-seeking ^90^Sr in food or potable water poses a big threat after nuclear accidents. The determination of ^90^Sr in environmental samples such as foodstuffs, however, requires a relatively time-consuming radiochemical separation as illustrated above, whereas γ-emitters can be quantified quickly. In order to still address the ^90^Sr issue properly to a dense network of samples taken from different regions, Japanese authorities assumed the intrinsic co-existence of radiostrontium together with γ- emitting radiocesium. As stated above, the activity of ^90^Sr was assumed to be 10% of the activity of ^137^Cs for the assessment of the regulatory limits. Obviously, this value originated from the experience with fallout radiostrontium and radiocesium from atmospheric nuclear explosions, for which the ^90^Sr:^137^Cs activity ratio of 1∶10 was reported e.g. in contaminated rice [33]. Our study indicates the correctness of this conservative approach: in no Japanese sample investigated herein, the ^90^Sr level was higher than 10% of the ^137^Cs level. In most samples, the ^137^Cs activity concentration was several orders of magnitude higher than the ^90^Sr the activity concentrations (see Figs. 2 and 3).

### Conclusions

Several hot spots in Japan were investigated with respect to the activity concentrations of β^−^ -emitting ^90^Sr and β^−^/γ-emitting ^134^Cs and ^137^Cs in soil and vegetation samples. Although the ^137^Cs activity levels were partly as high as in the kBq⋅g^−1^ range, the ^90^Sr contamination levels of any sample did not exceed the Bq⋅g^−1^ range. The radiocesium contamination could be clearly attributed to the Fukushima nuclear accident via its activity ratio fingerprint (^134^Cs/^137^Cs). Since short-lived ^89^Sr could no longer be determined, the source of the ^90^Sr theoretically could, in part, also be fallout from the nuclear explosions of the 20^th^ century or previous nuclear acidents. In any case, it is likely that releases from the Fukushima nuclear accident contributed much of the ^90^Sr that was measured at the hot spots.

The low contamination levels confirmed previous simulations by Schwantes et al. [18], who predicted that most of the radiostrontium was retained inside the reactors. In fact, the ^90^Sr activity concentrations were partly four orders of magnitude lower than the respective ^137^Cs activity concentrations.

The data set (though limited in terms of sample numbers) suggests an intrinsic coexistence of ^137^Cs and ^90^Sr in the contaminations caused by the Fukushima nuclear accident. This observation is of great importance for the current food monitoring campaigns, which currently rely on the assumption that the activity concentrations of β^−^-emitting ^90^Sr (which is relatively laborious to determine) is not higher than 10% of the level of γ-emitting ^137^Cs (which can be measured quickly). This assumption could be confirmed for the samples investigated herein.

## Supporting Information

Table S1 **Samples, sample locations and activity concentrations of the investigated radionuclides.** Uncertainties are due to counting statistics. Data were decay corrected to the time of the accident (11 March 2011).
(DOCX)
