# Supplementary material for: Concentration of Strontium-90 at Selected Hot Spots in Japan
Source: PLoS One. 2013 Mar 7;8(3):e57760. doi: 10.1371/journal.pone.0057760 (PMC3591386; doi:10.1371/journal.pone.0057760)
Supplement: Table S1 — Samples, sample locations and activity concentrations of the investigated radionuclides. Uncertainties are due to counting statistics. Data were decay corrected to the time of the accident (11 March 2011). (DOCX) [file pone.0057760.s001.docx]

**Table S1**

| Spot code (see Fig. 1) | Sample number | Sample type | Sample location | Coordinates (N, E) | Sampling date | Distance to damaged reactors | ^90^Sr, Bq/g | Uncertainty (%) | ^134^Cs, Bq/g | Uncertainty (%) | ^137^Cs, Bq/g | Uncertainty (%) | Activity ratio ^134^Cs/^137^Cs |
| --- | --- | --- | --- | --- | --- | --- | --- | --- | --- | --- | --- | --- | --- |
| A | A-S | soil | Main Gate Fukushima I | 37.417158,141.024714 | 2011-12-21 | 0.88 | 1.07 | 0.66 | 1720 | 1.2 | 1790 | 1.34 | 0.96 |
| A | A-V | vegetation | Main Gate Fukushima I | 37.417158,141.024714 | 2011-12-21 | 0.88 | 1.14 | 0.54 | 1240 | 0.52 | 1290 | 0.42 | 0.96 |
| B | B-S | soil | 1.5 km from Fukushima I | 37.417746,141.016817 | 2011-12-21 | 1.5 | 0.303 | 0.93 | 4350 | 0.23 | 4600 | 0.18 | 0.95 |
| B | B-V | vegetation | 1.5 km from Fukushima I | 37.417746,141.016817 | 2011-12-21 | 1.5 | 0.388 | 0.96 | 130 | 4.11 | 142 | 3.18 | 0.92 |
| C | C-S | soil | 1.9 km from Fukushima I | 37.417635,141.012247 | 2011-12-21 | 1.9 | 0.0318 | 1.21 | 122 | 0.95 | 127 | 0.41 | 0.96 |
| C | C-V | vegetation | 1.9 km from Fukushima I | 37.417635,141.012247 | 2011-12-21 | 1.9 | 0.448 | 0.96 | 223 | 0.68 | 237 | 0.56 | 0.94 |
| D | D-S | soil | 4.3 km from Fukushima I | 37.388743,141.008309 | 2011-12-21 | 4.3 | 0.232 | 0.9 | 2570 | 0.41 | 2740 | 0.41 | 0.94 |
| D | D-V | vegetation | 4.3 km from Fukushima I | 37.388743,141.008309 | 2011-12-21 | 4.3 | 0.253 | 1.01 | 2890 | 0.95 | 3120 | 0.73 | 0.93 |
| E | E-S | soil | Chimyo-ji temple | 37.495737,141.001373 | 2011-12-21 | 8.7 | 0.0297 | 1.24 | 23.5 | 3.19 | 25 | 1.12 | 0.94 |
| E | E-V | vegetation | Chimyo-ji temple | 37.495737,141.001373 | 2011-12-21 | 8.7 | 0.0261 | 1.25 | 15.8 | 3.87 | 18 | 0.88 | 0.88 |
| F | F-S | soil | Fukushima II NPP | 37.314889,141.014156 | 2011-12-21 | 12.0 | 0.268 | 0.84 | 20.3 | 1.25 | 21.7 | 0.89 | 0.94 |
| F | F-V | vegetation | Fukushima II NPP | 37.314889,141.014156 | 2011-12-21 | 12.0 | 0.0909 | 1.1 | 80.5 | 1.53 | 86.5 | 1.11 | 0.93 |
| G | G-S1 | soil | Odaka, minami-soma | 37.565875,140.992033 | 2011-12-21 | 16.4 | 0.067 | 1.1 | 59.4 | 2.1 | 62.2 | 0.86 | 0.95 |
| G | G-S2 | soil | Odaka, minami-soma | 37.565875,140.992033 | 2011-12-21 | 16.4 | 0.00886 | 1.25 | 8.5 | 5.12 | 9.17 | 1.4 | 0.93 |
| G | G-V | vegetation | Odaka, minami-soma | 37.565875,140.992033 | 2011-12-21 | 16.4 | 0.125 | 1.17 | 0.73 | 9.96 | 0.75 | 6.28 | 0.97 |
| H | H-S | soil | Sendai | 38.269,140.869 | 2012-07-20 | 95 | <0.003 |  | 4.3 | 7.33 | 5 | 5.39 | 0.86 |
| I | I-S ^a^ | soil | Kashiwa | 35.880008,139.98574 | 2011-10-26 | 195 | 0.035 | 1.21 | 406 | 0.7 | 421 | 0.36 | 0.96 |
| J | J-S1 ^b^ | soil | Yokohama | 35.54,139.63 | 2011-07-25 | 244 | 0.085 | 1.19 | 50 | 6.2 | 55 | 4.05 | 0.91 |
| J | J-S2 ^b^ | soil | Yokohama | 35.54,139.63 | 2011-07-25 | 244 | 0.0041 | 1.28 | 70.2 | 1.51 | 72.5 | 0.6 | 0.97 |

^a^ Sample I-S was collected on the roof of a building.

^b^ Samples J-S1 and J-S2 were collected in a drain.
